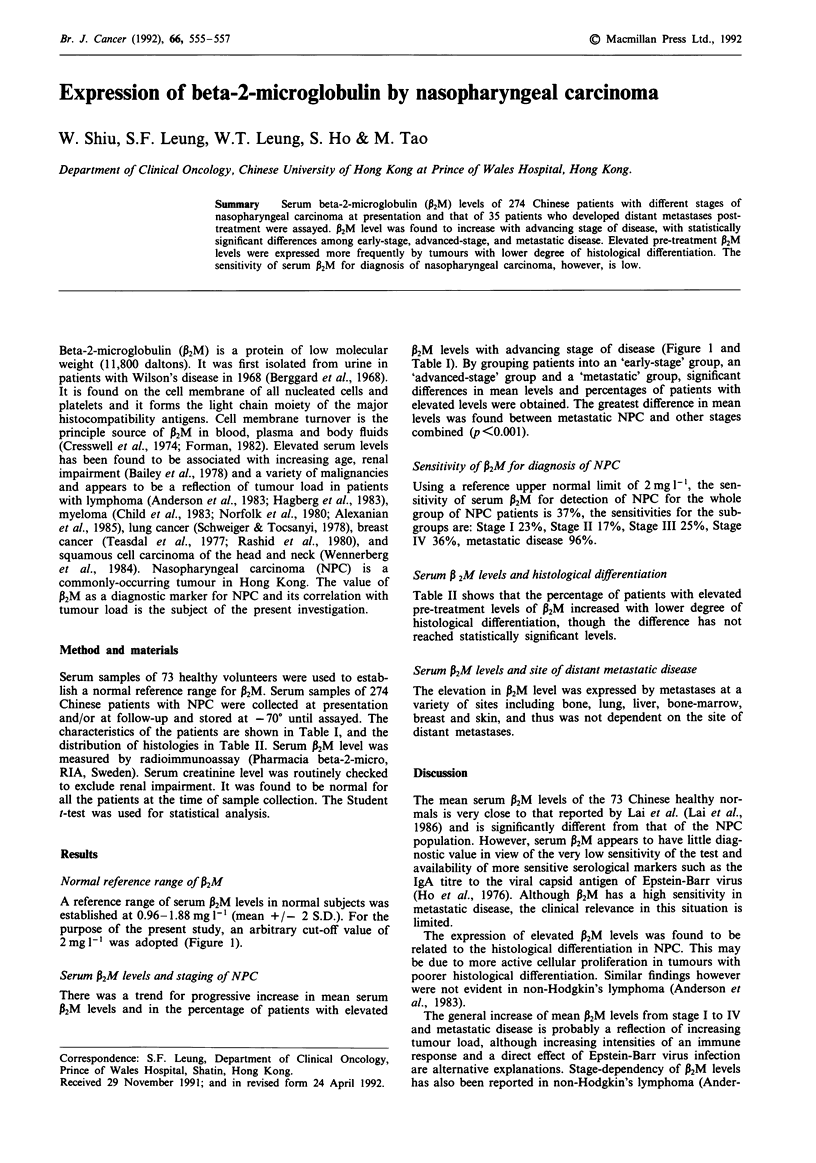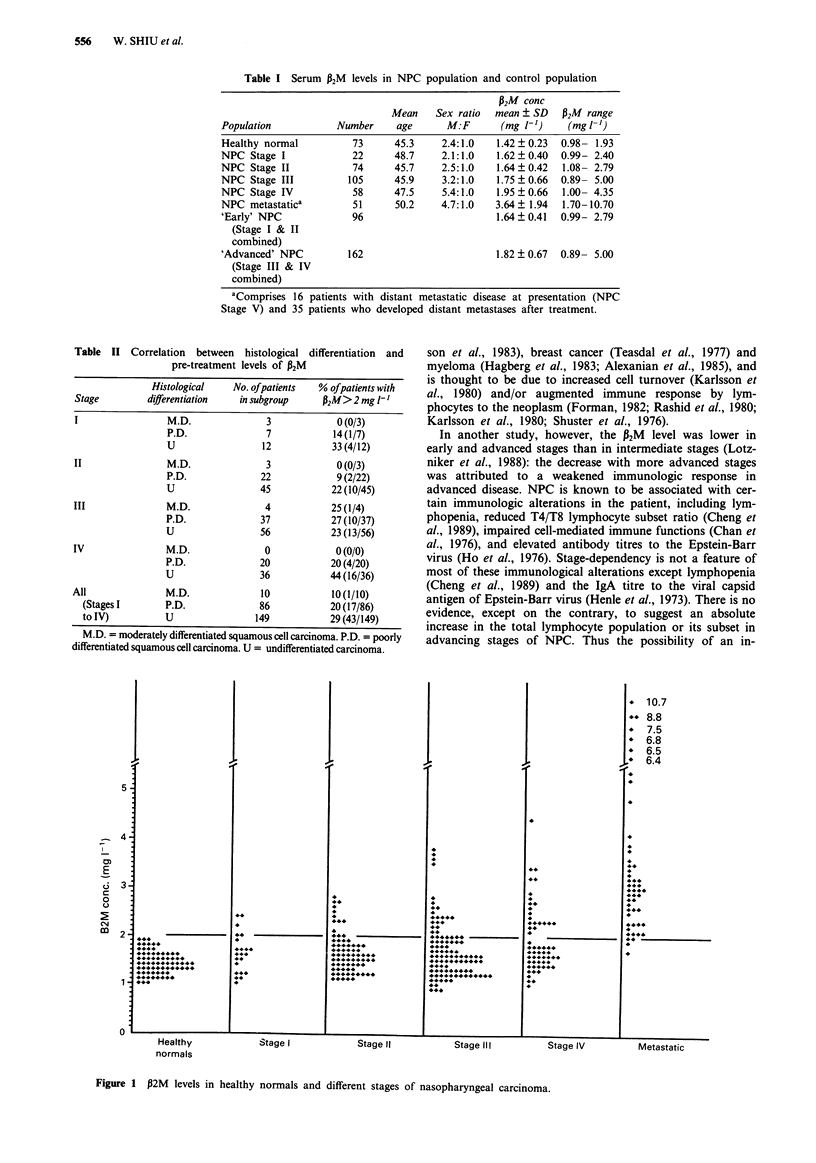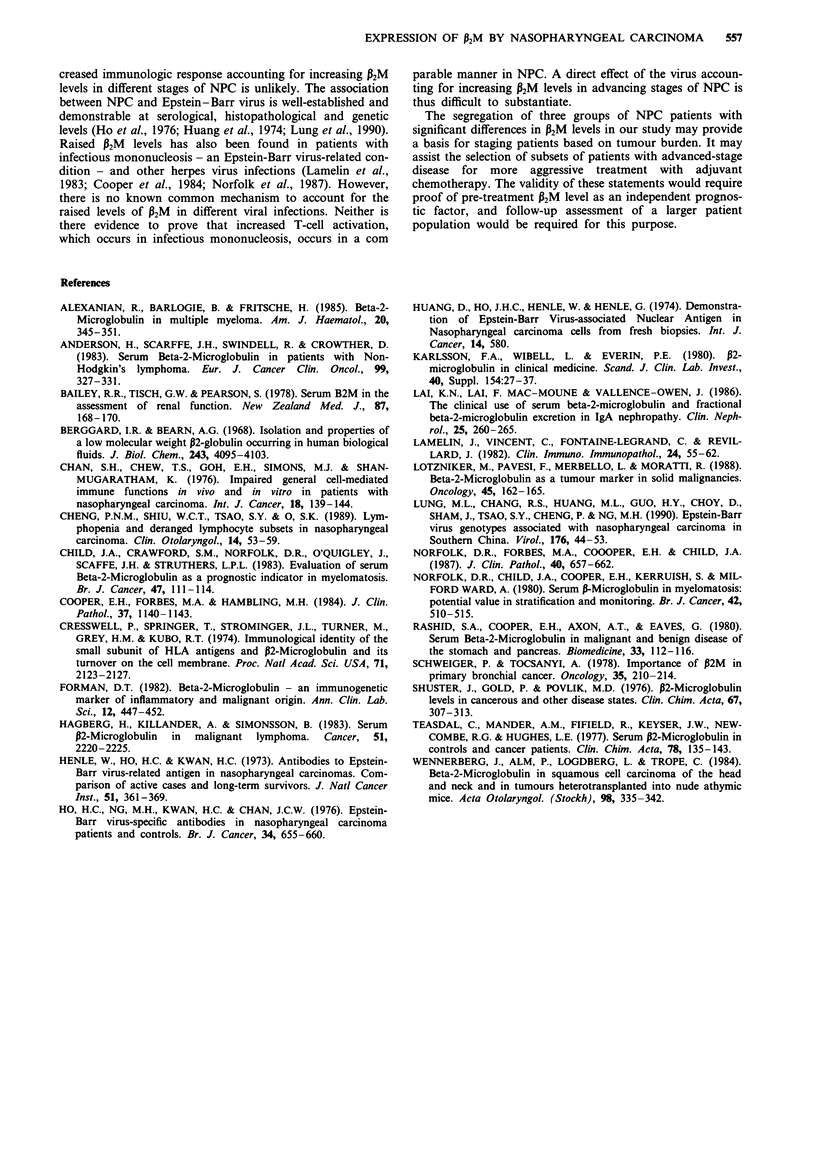# Expression of beta-2-microglobulin by nasopharyngeal carcinoma.

**DOI:** 10.1038/bjc.1992.313

**Published:** 1992-09

**Authors:** W. Shiu, S. F. Leung, W. T. Leung, S. Ho, M. Tao

**Affiliations:** Department of Clinical Oncology, Prince of Wales Hospital, Chinese University of Hong Kong.

## Abstract

Serum beta-2-microglobulin (beta 2M) levels of 274 Chinese patients with different stages of nasopharyngeal carcinoma at presentation and that of 35 patients who developed distant metastases post-treatment were assayed. beta 2M level was found to increase with advancing stage of disease, with statistically significant differences among early-stage, advanced-stage, and metastatic disease. Elevated pre-treatment beta 2M levels were expressed more frequently by tumours with lower degree of histological differentiation. The sensitivity of serum beta 2M for diagnosis of nasopharyngeal carcinoma, however, is low.


Br. J. Cancer (1992), 66, 555–557 © Macmillan Press Ltd., 1992

### Expression of beta-2-microglobulin by nasopharyngeal carcinoma

W. Shiu, S.F. Leung, W.T. Leung, S. Ho & M. Tao

*Department of Clinical Oncology, Chinese University of Hong Kong at Prince of Wales Hospital, Hong Kong.*

**Summary** Serum beta-2-microglobulin ($\beta_2M$) levels of 274 Chinese patients with different stages of nasopharyngeal carcinoma at presentation and that of 35 patients who developed distant metastases post-treatment were assayed. $\beta_2M$ level was found to increase with advancing stage of disease, with statistically significant differences among early-stage, advanced-stage, and metastatic disease. Elevated pre-treatment $\beta_2M$ levels were expressed more frequently by tumours with lower degree of histological differentiation. The sensitivity of serum $\beta_2M$ for diagnosis of nasopharyngeal carcinoma, however, is low.

Beta-2-microglobulin ($\beta_2M$) is a protein of low molecular weight (11,800 daltons). It was first isolated from urine in patients with Wilson's disease in 1968 (Berggard *et al.*, 1968). It is found on the cell membrane of all nucleated cells and platelets and it forms the light chain moiety of the major histocompatibility antigens. Cell membrane turnover is the principle source of $\beta_2M$ in blood, plasma and body fluids (Cresswell *et al.*, 1974; Forman, 1982). Elevated serum levels has been found to be associated with increasing age, renal impairment (Bailey *et al.*, 1978) and a variety of malignancies and appears to be a reflection of tumour load in patients with lymphoma (Anderson *et al.*, 1983; Hagberg *et al.*, 1983), myeloma (Child *et al.*, 1983; Norfolk *et al.*, 1980; Alexanian *et al.*, 1985), lung cancer (Schweiger & Tocsanyi, 1978), breast cancer (Teasdal *et al.*, 1977; Rashid *et al.*, 1980), and squamous cell carcinoma of the head and neck (Wennerberg *et al.*, 1984). Nasopharyngeal carcinoma (NPC) is a commonly-occurring tumour in Hong Kong. The value of $\beta_2M$ as a diagnostic marker for NPC and its correlation with tumour load is the subject of the present investigation.

### Method and materials

Serum samples of 73 healthy volunteers were used to establish a normal reference range for $\beta_2M$. Serum samples of 274 Chinese patients with NPC were collected at presentation and/or at follow-up and stored at −70° until assayed. The characteristics of the patients are shown in Table I, and the distribution of histologies in Table II. Serum $\beta_2M$ level was measured by radioimmunoassay (Pharmacia beta-2-micro, RIA, Sweden). Serum creatinine level was routinely checked to exclude renal impairment. It was found to be normal for all the patients at the time of sample collection. The Student *t*-test was used for statistical analysis.

### Results

### *Normal reference range of $\beta_2M$*

A reference range of serum $\beta_2M$ levels in normal subjects was established at 0.96–1.88 $mg\,l^{-1}$ (mean +/− 2 S.D.). For the purpose of the present study, an arbitrary cut-off value of 2 $mg\,l^{-1}$ was adopted (Figure 1).

### *Serum $\beta_2M$ levels and staging of NPC*

There was a trend for progressive increase in mean serum $\beta_2M$ levels and in the percentage of patients with elevated $\beta_2M$ levels with advancing stage of disease (Figure 1 and Table I). By grouping patients into an 'early-stage' group, an 'advanced-stage' group and a 'metastatic' group, significant differences in mean levels and percentages of patients with elevated levels were obtained. The greatest difference in mean levels was found between metastatic NPC and other stages combined ($p<0.001$).

### *Sensitivity of $\beta_2M$ for diagnosis of NPC*

Using a reference upper normal limit of 2 $mg\,l^{-1}$, the sensitivity of serum $\beta_2M$ for detection of NPC for the whole group of NPC patients is 37%, the sensitivities for the subgroups are: Stage I 23%, Stage II 17%, Stage III 25%, Stage IV 36%, metastatic disease 96%.

### *Serum $\beta_2M$ levels and histological differentiation*

Table II shows that the percentage of patients with elevated pre-treatment levels of $\beta_2M$ increased with lower degree of histological differentiation, though the difference has not reached statistically significant levels.

### *Serum $\beta_2M$ levels and site of distant metastatic disease*

The elevation in $\beta_2M$ level was expressed by metastases at a variety of sites including bone, lung, liver, bone-marrow, breast and skin, and thus was not dependent on the site of distant metastases.

### Discussion

The mean serum $\beta_2M$ levels of the 73 Chinese healthy normals is very close to that reported by Lai *et al.* (Lai *et al.*, 1986) and is significantly different from that of the NPC population. However, serum $\beta_2M$ appears to have little diagnostic value in view of the very low sensitivity of the test and availability of more sensitive serological markers such as the IgA titre to the viral capsid antigen of Epstein-Barr virus (Ho *et al.*, 1976). Although $\beta_2M$ has a high sensitivity in metastatic disease, the clinical relevance in this situation is limited.

The expression of elevated $\beta_2M$ levels was found to be related to the histological differentiation in NPC. This may be due to more active cellular proliferation in tumours with poorer histological differentiation. Similar findings however were not evident in non-Hodgkin's lymphoma (Anderson *et al.*, 1983).

The general increase of mean $\beta_2M$ levels from stage I to IV and metastatic disease is probably a reflection of increasing tumour load, although increasing intensities of an immune response and a direct effect of Epstein-Barr virus infection are alternative explanations. Stage-dependency of $\beta_2M$ levels has also been reported in non-Hodgkin's lymphoma (Ander-

Correspondence: S.F. Leung, Department of Clinical Oncology, Prince of Wales Hospital, Shatin, Hong Kong.
Received 29 November 1991; and in revised form 24 April 1992.

**Table I** Serum $\beta_2$M levels in NPC population and control population

| *Population* | *Number* | *Mean age* | *Sex ratio M:F* | *$\beta_2$M conc mean ± SD (mg $l^{-1}$)* | *$\beta_2$M range (mg $l^{-1}$)* |
|---|---|---|---|---|---|
| Healthy normal | 73 | 45.3 | 2.4:1.0 | 1.42 ± 0.23 | 0.98- 1.93 |
| NPC Stage I | 22 | 48.7 | 2.1:1.0 | 1.62 ± 0.40 | 0.99- 2.40 |
| NPC Stage II | 74 | 45.7 | 2.5:1.0 | 1.64 ± 0.42 | 1.08- 2.79 |
| NPC Stage III | 105 | 45.9 | 3.2:1.0 | 1.75 ± 0.66 | 0.89- 5.00 |
| NPC Stage IV | 58 | 47.5 | 5.4:1.0 | 1.95 ± 0.66 | 1.00- 4.35 |
| NPC metastatic[a] | 51 | 50.2 | 4.7:1.0 | 3.64 ± 1.94 | 1.70-10.70 |
| 'Early' NPC (Stage I & II combined) | 96 | | | 1.64 ± 0.41 | 0.99- 2.79 |
| 'Advanced' NPC (Stage III & IV combined) | 162 | | | 1.82 ± 0.67 | 0.89- 5.00 |

[a]Comprises 16 patients with distant metastatic disease at presentation (NPC Stage V) and 35 patients who developed distant metastases after treatment.

**Table II** Correlation between histological differentiation and pre-treatment levels of $\beta_2$M

| *Stage* | *Histological differentiation* | *No. of patients in subgroup* | *% of patients with $\beta_2M > 2$ mg $l^{-1}$* |
|---|---|---|---|
| I | M.D. | 3 | 0 (0/3) |
| | P.D. | 7 | 14 (1/7) |
| | U | 12 | 33 (4/12) |
| II | M.D. | 3 | 0 (0/3) |
| | P.D. | 22 | 9 (2/22) |
| | U | 45 | 22 (10/45) |
| III | M.D. | 4 | 25 (1/4) |
| | P.D. | 37 | 27 (10/37) |
| | U | 56 | 23 (13/56) |
| IV | M.D. | 0 | 0 (0/0) |
| | P.D. | 20 | 20 (4/20) |
| | U | 36 | 44 (16/36) |
| All (Stages I to IV) | M.D. | 10 | 10 (1/10) |
| | P.D. | 86 | 20 (17/86) |
| | U | 149 | 29 (43/149) |

M.D. = moderately differentiated squamous cell carcinoma. P.D. = poorly differentiated squamous cell carcinoma. U = undifferentiated carcinoma.

son *et al.*, 1983), breast cancer (Teasdal *et al.*, 1977) and myeloma (Hagberg *et al.*, 1983; Alexanian *et al.*, 1985), and is thought to be due to increased cell turnover (Karlsson *et al.*, 1980) and/or augmented immune response by lymphocytes to the neoplasm (Forman, 1982; Rashid *et al.*, 1980; Karlsson *et al.*, 1980; Shuster *et al.*, 1976).

In another study, however, the $\beta_2$M level was lower in early and advanced stages than in intermediate stages (Lotzniker *et al.*, 1988): the decrease with more advanced stages was attributed to a weakened immunologic response in advanced disease. NPC is known to be associated with certain immunologic alterations in the patient, including lymphopenia, reduced T4/T8 lymphocyte subset ratio (Cheng *et al.*, 1989), impaired cell-mediated immune functions (Chan *et al.*, 1976), and elevated antibody titres to the Epstein-Barr virus (Ho *et al.*, 1976). Stage-dependency is not a feature of most of these immunological alterations except lymphopenia (Cheng *et al.*, 1989) and the IgA titre to the viral capsid antigen of Epstein-Barr virus (Henle *et al.*, 1973). There is no evidence, except on the contrary, to suggest an absolute increase in the total lymphocyte population or its subset in advancing stages of NPC. Thus the possibility of an in-

**Figure 1** β2M levels in healthy normals and different stages of nasopharyngeal carcinoma.

creased immunologic response accounting for increasing $\beta_2$M levels in different stages of NPC is unlikely. The association between NPC and Epstein–Barr virus is well-established and demonstrable at serological, histopathological and genetic levels (Ho *et al.*, 1976; Huang *et al.*, 1974; Lung *et al.*, 1990). Raised $\beta_2$M levels has also been found in patients with infectious mononucleosis – an Epstein-Barr virus-related condition – and other herpes virus infections (Lamelin *et al.*, 1983; Cooper *et al.*, 1984; Norfolk *et al.*, 1987). However, there is no known common mechanism to account for the raised levels of $\beta_2$M in different viral infections. Neither is there evidence to prove that increased T-cell activation, which occurs in infectious mononucleosis, occurs in a com parable manner in NPC. A direct effect of the virus accounting for increasing $\beta_2$M levels in advancing stages of NPC is thus difficult to substantiate.

The segregation of three groups of NPC patients with significant differences in $\beta_2$M levels in our study may provide a basis for staging patients based on tumour burden. It may assist the selection of subsets of patients with advanced-stage disease for more aggressive treatment with adjuvant chemotherapy. The validity of these statements would require proof of pre-treatment $\beta_2$M level as an independent prognostic factor, and follow-up assessment of a larger patient population would be required for this purpose.

### References

ALEXANIAN, R., BARLOGIE, B. & FRITSCHE, H. (1985). Beta-2-Microglobulin in multiple myeloma. *Am. J. Haematol.*, **20**, 345–351.

ANDERSON, H., SCARFFE, J.H., SWINDELL, R. & CROWTHER, D. (1983). Serum Beta-2-Microglobulin in patients with Non-Hodgkin's lymphoma. *Eur. J. Cancer Clin. Oncol.*, **99**, 327–331.

BAILEY, R.R., TISCH, G.W. & PEARSON, S. (1978). Serum B2M in the assessment of renal function. *New Zealand Med. J.*, **87**, 168–170.

BERGGARD, I.R. & BEARN, A.G. (1968). Isolation and properties of a low molecular weight β2-globulin occurring in human biological fluids. *J. Biol. Chem.*, **243**, 4095–4103.

CHAN, S.H., CHEW, T.S., GOH, E.H., SIMONS, M.J. & SHANMUGARATHAM, K. (1976). Impaired general cell-mediated immune functions *in vivo* and *in vitro* in patients with nasopharyngeal carcinoma. *Int. J. Cancer*, **18**, 139–144.

CHENG, P.N.M., SHIU, W.C.T., TSAO, S.Y. & O, S.K. (1989). Lymphopenia and deranged lymphocyte subsets in nasopharyngeal carcinoma. *Clin. Otolaryngol.*, **14**, 53–59.

CHILD, J.A., CRAWFORD, S.M., NORFOLK, D.R., O'QUIGLEY, J., SCAFFE, J.H. & STRUTHERS, L.P.L. (1983). Evaluation of serum Beta-2-Microglobulin as a prognostic indicator in myelomatosis. *Br. J. Cancer*, **47**, 111–114.

COOPER, E.H., FORBES, M.A. & HAMBLING, M.H. (1984). *J. Clin. Pathol.*, **37**, 1140–1143.

CRESSWELL, P., SPRINGER, T., STROMINGER, J.L., TURNER, M., GREY, H.M. & KUBO, R.T. (1974). Immunological identity of the small subunit of HLA antigens and β2-Microglobulin and its turnover on the cell membrane. *Proc. Natl Acad. Sci. USA*, **71**, 2123–2127.

FORMAN, D.T. (1982). Beta-2-Microglobulin – an immunogenetic marker of inflammatory and malignant origin. *Ann. Clin. Lab. Sci.*, **12**, 447–452.

HAGBERG, H., KILLANDER, A. & SIMONSSON, B. (1983). Serum β2-Microglobulin in malignant lymphoma. *Cancer*, **51**, 2220–2225.

HENLE, W., HO, H.C. & KWAN, H.C. (1973). Antibodies to Epstein-Barr virus-related antigen in nasopharyngeal carcinomas. Comparison of active cases and long-term survivors. *J. Natl Cancer Inst.*, **51**, 361–369.

HO, H.C., NG, M.H., KWAN, H.C. & CHAN, J.C.W. (1976). Epstein-Barr virus-specific antibodies in nasopharyngeal carcinoma patients and controls. *Br. J. Cancer*, **34**, 655–660.

HUANG, D., HO, J.H.C., HENLE, W. & HENLE, G. (1974). Demonstration of Epstein-Barr Virus-associated Nuclear Antigen in Nasopharyngeal carcinoma cells from fresh biopsies. *Int. J. Cancer*, **14**, 580.

KARLSSON, F.A., WIBELL, L. & EVERIN, P.E. (1980). β2-microglobulin in clinical medicine. *Scand. J. Clin. Lab. Invest.*, **40**, Suppl. 154:27–37.

LAI, K.N., LAI, F. MAC-MOUNE & VALLENCE-OWEN, J. (1986). The clinical use of serum beta-2-microglobulin and fractional beta-2-microglobulin excretion in IgA nephropathy. *Clin. Nephrol.*, **25**, 260–265.

LAMELIN, J., VINCENT, C., FONTAINE-LEGRAND, C. & REVILLARD, J. (1982). *Clin. Immuno. Immunopathol.*, **24**, 55–62.

LOTZNIKER, M., PAVESI, F., MERBELLO, L. & MORATTI, R. (1988). Beta-2-Microglobulin as a tumour marker in solid malignancies. *Oncology*, **45**, 162–165.

LUNG, M.L., CHANG, R.S., HUANG, M.L., GUO, H.Y., CHOY, D., SHAM, J., TSAO, S.Y., CHENG, P. & NG, M.H. (1990). Epstein-Barr virus genotypes associated with nasopharyngeal carcinoma in Southern China. *Virol.*, **176**, 44–53.

NORFOLK, D.R., FORBES, M.A., COOOPER, E.H. & CHILD, J.A. (1987). *J. Clin. Pathol.*, **40**, 657–662.

NORFOLK, D.R., CHILD, J.A., COOPER, E.H., KERRUISH, S. & MILFORD WARD, A. (1980). Serum β-Microglobulin in myelomatosis: potential value in stratification and monitoring. *Br. J. Cancer*, **42**, 510–515.

RASHID, S.A., COOPER, E.H., AXON, A.T., & EAVES, G. (1980). Serum Beta-2-Microglobulin in malignant and benign disease of the stomach and pancreas. *Biomedicine*, **33**, 112–116.

SCHWEIGER, P. & TOCSANYI, A. (1978). Importance of β2M in primary bronchial cancer. *Oncology*, **35**, 210–214.

SHUSTER, J., GOLD, P. & POVLIK, M.D. (1976). β2-Microglobulin levels in cancerous and other disease states. *Clin. Chim. Acta*, **67**, 307–313.

TEASDAL, C., MANDER, A.M., FIFIELD, R., KEYSER, J.W., NEWCOMBE, R.G. & HUGHES, L.E. (1977). Serum β2-Microglobulin in controls and cancer patients. *Clin. Chim. Acta*, **78**, 135–143.

WENNERBERG, J., ALM, P., LOGDBERG, L. & TROPE, C. (1984). Beta-2-Microglobulin in squamous cell carcinoma of the head and neck and in tumours heterotransplanted into nude athymic mice. *Acta Otolaryngol. (Stockh)*, **98**, 335–342.